# *Trypanosoma brucei*: trypanocidal and cell swelling activities of lasalocid acid

**DOI:** 10.1007/s00436-017-5624-6

**Published:** 2017-09-27

**Authors:** Dietmar Steverding, Adam Huczyński

**Affiliations:** 1 Bob Champion Research & Education Building, Norwich Medical School, University of East Anglia, Norwich, UK; 2 Faculty of Chemistry, Adam Mickiewicz University, Poznań, Poland

**Keywords:** African trypanosomiasis, *Trypanosoma brucei*, Lasalocid acid, Polyether ionophores

## Abstract

Chemotherapeutic treatment of human and animal trypanosomiasis is unsatisfactory because only a few drugs are available. As these drugs have poor efficacy and cause adverse reactions, more effective and tolerable medications are needed. As the polyether ionophore antibiotic lasalocid acid is used as medicated feed additive in cattle, the compound was tested for its trypanocidal and cytotoxic activity against bloodstream forms of *Trypanosoma brucei* and human myeloid HL-60 cells. The concentrations required of lasalocid acid to reduce the growth rate of trypanosomes by 50% and to kill the parasites were 1.75 and 10 μM, respectively. The ionophore displayed also cytotoxic activity against HL-60 cells but the human cells were about 10 to 14 times less sensitive indicating moderate selectivity. As the trypanocidal mechanism of action of polyether ionophore antibiotics is due to a sodium influx-induced cell swelling, the effect of lasalocid acid on cell volume change in bloodstream-form trypanosomes was investigated. Interestingly, lasalocid acid induced a much faster cell swelling in trypanosomes than the more trypanocidal related ionophore salinomycin. These results support further investigations of lasalocid acid and derivatives thereof as potential agents against African trypanosomiasis.

**Electronic supplementary material:**

The online version of this article (10.1007/s00436-017-5624-6) contains supplementary material, which is available to authorized users.

## Introduction

African trypanosomiasis is an infectious parasitic disease of humans (sleeping sickness) and livestock (nagana disease) of similar aetiology and epidemiology. The causative agents of the diseases are flagellated protozoans of the genus *Trypanosoma*. The parasites are transmitted by the bite of infected tsetse flies (*Glossina* sp.) and live and multiply in the blood and tissue fluids of their mammalian host. The distribution of trypanosomiasis in Africa corresponds to the range of tsetse flies and comprises an area of 8 million km^2^ between 14°N and 20°S latitude, a region known as the tsetse belt (Steverding 2017). African trypanosomiasis has severely repressed the economic and cultural development of central Africa in the past (Steverding 2008) and still continues to cause morbidity, mortality and economic deprivation in sub-Saharan Africa (Steverding 2017).

Only a few drugs are currently available for chemotherapy of African trypanosomiasis (Holmes et al. 2004; Steverding 2010). All these drugs have major drawbacks including poor efficacy, significant toxicity and requirement for parenteral administration, and are being increasingly subject to drug resistance (Matovu et al. 2001; Fairlamb 2003; Delespaux and de Koning 2007). Hence, effective and better tolerated chemotherapies are urgently needed for treatment of African trypanosomiasis.

**Fig. 1:**
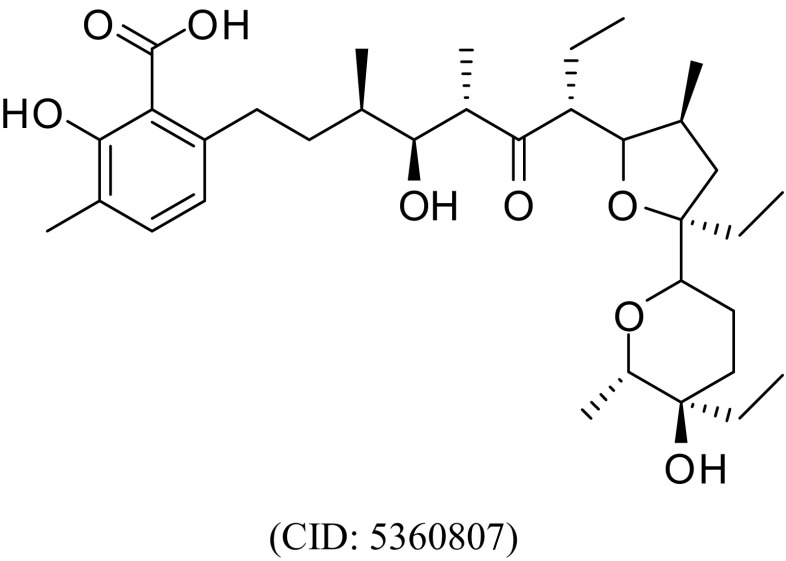
Structure of lasalocid acid. The PubChem compound identifier (CID) for the compound is shown in parentheses

Lasalocid acid (Fig. 1) is a polyether ionophore antibiotic produced by strains of the bacterium *Streptomyces lasaliensis*. It is used in cattle as medicated feed additive (Bovatec®) to improve feed efficiency and to increase the rate of weight gain, and to control coccidiosis caused by *Eimeria bovis* and *Eimeria* *zuernii* (Flanders and Gillespie 2016). In addition, lasalocid acid like other polyether ionophore antibiotics is commercially used as anti-coccidial drugs for the prevention and control of *Eimeria* infections in poultry (Kant et al. 2013). The related compound salinomycin has been shown in clinical pilot studies to be able to eliminate cancer stem cells and to induce partial clinical regression of heavily pretreated and therapy-resistant cancers in patients demonstrating the in vivo activity of polyether ionophore antibiotics (Naujokat and Steinhart 2012). These facts in connection with previous findings that other polyether ionophore antibiotics (salinomycin and monensin) display promising trypanocidal activities (Steverding and Sexton 2013; Steverding et al. 2016) prompted us to investigate the antitrypanosomal action of lasalocid acid and to provide a proof of concept of the potential use of this compound as trypanocide.

## Materials and methods

### Lasalocid acid

Lasalocid acid was purified from its sodium salt that was isolated from Avatec Premix as previously described (Huczyński et al. 2013).

### Cell culture

Bloodstream forms of *T. brucei* (clone 427-221a; Hirumi et al. 1980) and human myeloid leukaemia HL-60 cells (Collins et al. 1977) were maintained in Baltz medium (Baltz et al. 1985) supplemented with 16.7% heat-inactivated foetal bovine serum in a humidified atmosphere containing 5% CO_2_ at 37 °C.

### In vitro toxicity assay

Toxicity assays were carried out as previously described (Merschjohann et al. 2001) with some modifications. In brief, cells (trypanosomes and HL-60 cells) were seeded in 96 well plates in a final volume of 200 μl of the Baltz medium containing various concentrations of test compounds (tenfold dilution from 100 μM to 10 nM) and 1% DMSO. Wells just containing medium and 1% DMSO served as controls. The initial cell densities were 1 × 10^4^/ml for trypanosomes and 5 × 10^4^/ml for HL-60 cells. After 24 h incubation, 20 μl of a 0.5 mM resazurin solution prepared in PBS was added and the cells were incubated for a further 48 h. Thereafter, the absorbance of wells was read on a BioTek ELx808 microplate reader using a test wavelength of 570 nm and a reference wavelength of 630 nm. The 50% growth inhibition (GI_50_) value, i.e. the concentration of a compound necessary to reduce the growth rate of cells by 50% compared to the control was determined by linear interpolation (Huber and Koella 1993). The minimum inhibitory concentration (MIC) values, i.e. the concentration of the drug at which all trypanosomes and human cells were killed, were determined microscopically.

### Swelling experiment

Cell volume can be measured by light scattering which has been used to monitor volume change in many different cell types including bacteria, mammalian cells and protozoans. For *Giardia intestinalis*, a flagellated protozoan parasite like *T. brucei*, the absorbance of cell suspensions have been shown to be similar between 450 and 550 nm (Park et al. 1997). Based on the available filters of the BioTek ELx808 microplate reader, we determined changes in cell volume of trypanosomes at 490 nm as previously described (Steverding and Sexton 2013). In brief, bloodstream forms of *T. brucei* were seeded at a density of 5 × 10^7^ cells/ml in 96 well plates in a final volume of 200 μl Baltz medium containing 100 μM ionophore and 1% DMSO (test) or 1% DMSO alone (control). Additionally, some swelling experiments were performed in the presence of 6 mM of the divalent metal-chelating agent EDTA. Absorbance of the cultures was measured every 10 min. A decrease in absorbance corresponds to an increase in cell volume.

## Results and discussion

**Fig. 2:**
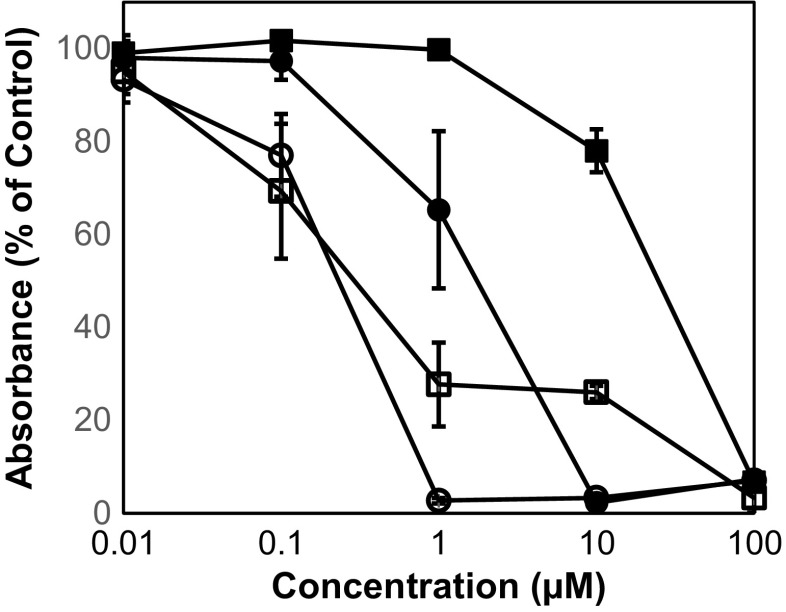
Effect of lasalocid acid and salinomycin on the growth of bloodstream forms of *T. brucei* and human myeloid leukaemia HL-60 cell. Trypanosomes (circles) and HL-60 cells (squares) were incubated with varying concentration of lasalocid acid (closed symbols) or salinomycin (open symbols). After 72 h of culture, cell viability and proliferation were determined with the colorimetric dye resazurin. The experiment was repeated three times and mean values ± SD of three experiments are shown

Lasalocid acid showed a dose-dependent inhibitory effect on the growth of bloodstream forms of *T. brucei* with a MIC value of 10 μM and a GI_50_ value of 1.75 μM (Fig. 2). Compared with salinomycin, lasalocid acid was 7.6 and 10 times less trypanocidal (MIC and GI_50_ values for salinomycin were determined to be 1 and 0.23 μM, respectively (Fig. 2)). On the other hand, lasalocid acid was less cytotoxic towards HL-60 cells than salinomycin, the corresponding MIC and GI_50_ values being 100 and 24.7 μM for lasalocid acid and 1 and 0.32 μM for salinomyicn, respectively (Fig. 2). Thus, while salinomycin showed no selectivity (cytotoxic to trypanocidal activity ratio) with MIC and GI_50_ ratios of 1 and 1.4, lasalocid acid exhibited moderated selectivity with indices of ≥ 10. The unfavourable selectivity of salinomycin can be attributed to its high cytotoxicity towards HL-60 cells, with our determined GI_50_ value of 0.32 μM being in good agreement with previously reported values of 0.29–0.44 μM (Huczyński et al. 2012, 2015; Steverding and Sexton 2013; Antoszczak et al. 2014; Steverding et al. 2016).

**Fig. 3:**
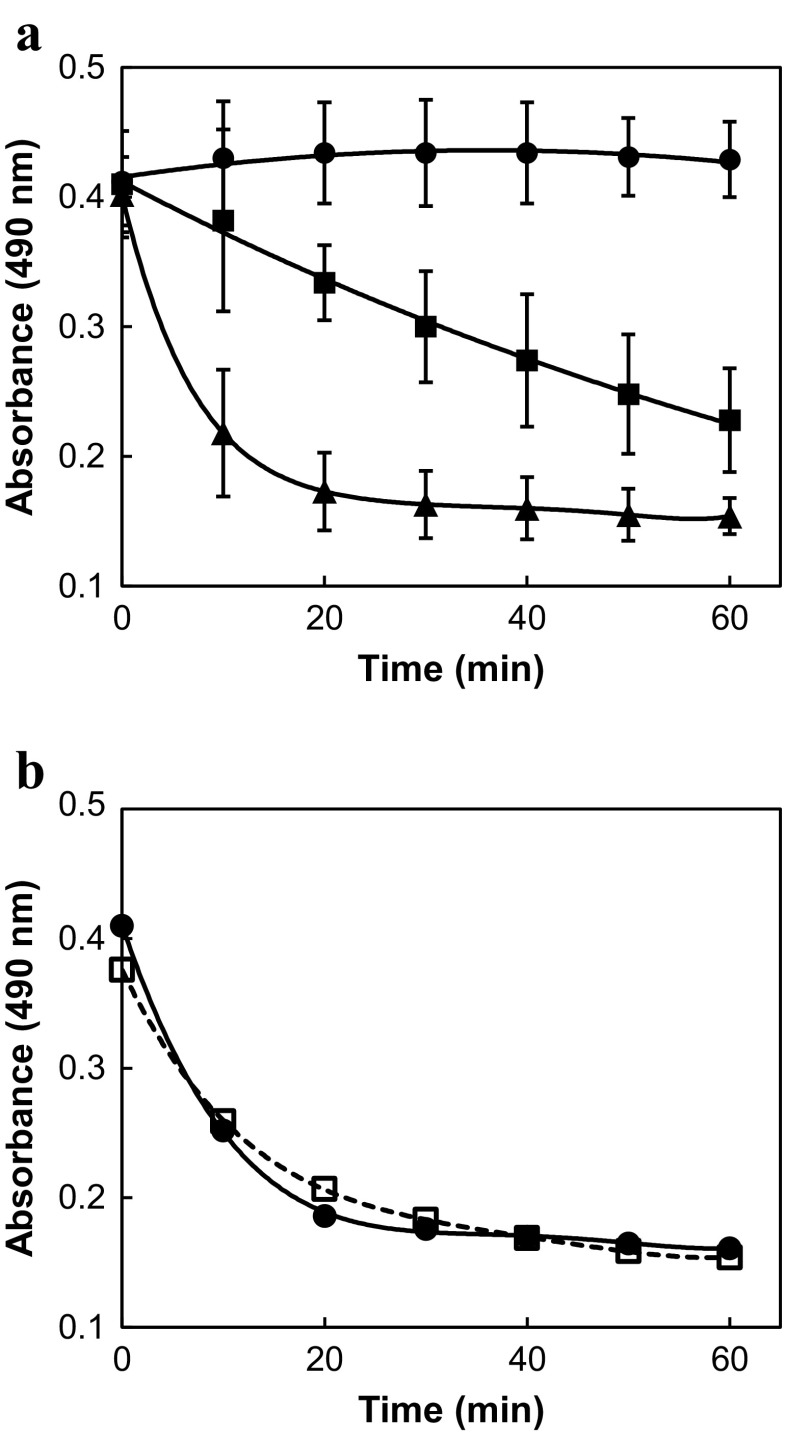
Effect of polyether ionophore antibiotics on the cell volume of bloodstream forms of *T. brucei*. **a** Trypanosomes (5 × 10^7^ cell/ml) were incubated with 100 μM lasalocid acid (triangles) or salinomycin (squares) in Baltz medium in the presence of 1% DMSO. Controls (circles) were incubated with 1% DMSO. Every 10 min, the absorbance at 490 nm was measured. Mean values ± SD of three experiments are shown. Except for the time point 0 min at all other time points, the absorbance values were statistically significantly different from each other (One-way ANOVA test, *p* < 0.01). **b** Trypanosomes (5 × 10^7^ cell/ml) were incubated with 100 μM lasalocid acid in the absence (closed circles, solid line) or presence of 6 mM EDTA (open squares, dashed line) in Baltz medium containing 1% DMSO. Every 10 min, the absorbance at 490 nm was measured. Mean values of three experiments are shown. For clarity, the standard deviations were omitted. The standard deviations ranged between 17.5–25.1% of the mean values. At each time point, the data points of the two curves were statistically not significantly different (*p* = 0.465–0.977, Student’s *t* test)

The biological activity of polyether ionophore antibiotics is due to initiation of an increase in the intracellular concentration of Na^+^ cations by their ability to transport these ions across biological membranes (Pressman et al. 1980). In cancer cells, this influx of Na^+^ cations seems to be responsible for the induction of apoptosis (Huczyński 2012) while in bloodstream-form trypanosomes, it leads to swelling of the cell by subsequent entry of water (Steverding and Sexton 2013). In addition, the rate of swelling in trypanosomes seems to be depending on the trypanocidal activity of the ionophore: the higher the trypanocidal activity, the faster the swelling (Steverding et al. 2016). Given that lasalocid acid is less trypanocidal than salinomycin, it was interesting to test whether lasalocid acid would cause a slower swelling rate compared to salinomycin. To be able to record measurable changes in absorbance, a high cell density (5 × 10^7^ cells/ml) and a high ionophore concentration (100 μM) are required (Steverding et al. 2016). Surprisingly, trypanosomes incubated with lasalocid acid swelled much faster than parasites treated with salinomycin (Fig. 3a). Already, after 20 min incubation, no further swelling was recorded indicating that the endpoint of the swelling process was already reached by which the trypanosomes started to die. In contrast, trypanosomes exposed to salinomycin continued to swell until the end of the experiment and started to die after 50 min of incubation. An explanation for the fast swelling activity of lasalocid acid may be the ability of the compound to transport Ca^2+^ cations across membranes that other polyether ionophore antibiotics lack (Pressman et al. 1980). In particular, the large Ca^2+^ concentration gradient of 20,000 (extracellular ~ 2 mM, intracellular ~ 100 nM; Ruben et al. 1991) would be more than sufficient to induce an ionophore-driven influx of Ca^2+^-ions that could cause a rapid swelling of trypanosomes. For comparison, the Na^+^ concentration gradient is just about 10 (extracellular 144 mM, intracellular 13.7 mM; Nolan and Voorheis 2000). In order to test whether the Ca^2+^ transport activity of lasalocid acid is indeed, the reason for the observed prompt swelling of trypanosomes, a swelling experiment was carried out in the presence of 6 mM of EDTA. This chelating agent has a much higher binding affinity for Ca^2+^-ions than lasalocid acid (*K*_S_ values for the Ca^2+^-complexes of EDTA and lasalocid acid are 10^7.9^ (estimated for pH 7.5) and 10^2.57^ (in methanol; Degani and Friedman 1974), respectively). As EDTA binds Mg^2+^ cations as well and as the combined concentration of Ca^2+^ and Mg^2+^ in the Baltz medium is approximately 3 mM, the employed concentration of the chelating agent of 6 mM was determined to be sufficient to reduce the extracellular Ca^2+^ concentration below 10 nM, and thus significantly below the intracellular Ca^2+^ concentration of bloodstream forms of *T. brucei*. Under these conditions, any ionophore-driven Ca^2+^-transport would be in the efflux direction. However, no difference in the swelling rate of the parasites upon addition of lasalocid acid in the presence or absence of 6 mM EDTA was observed (Fig. 3b). Hence, any Ca^2+^-transport across the plasma membrane seems not to play any role in the lasalocid acid-induced swelling of bloodstream forms of *T. brucei*, and that the observed fast swelling is solely due to the influx of Na^+^ cations. The reason why lasalocid acid and salinomycin differ in their swelling rates and trypanocidal activities may be just due to their affinity for Na^+^. While the lower *K*_S_ value of lasalocid acid for Na^+^ cations (10^2.57^; Degani and Friedman 1974) favours an easier transport of the ion across membranes, the higher *K*_S_ value of salinomycin for Na^+^ cations (10^3.31^; Pressman et al. 1980) facilitates a higher trypanocidal activity.

As chemical modification can increase the trypanocidal activity of polyether ionophore antibiotics (Steverding et al. 2016), we also studied the antitrypanosomal effect of seven Mannich base derivatives of lasalocid acid. The synthesis of the Mannich base derivatives tested is described elsewhere (Huczyński et al. 2013). However, none of the derivatives displayed better trypanocidal activity and selectivity than the parent compound lasalocid acid (Supplementary Table S1). This observation indicates that modification of the carboxyl group of lasalocid acid by Mannich base alkyl/aryl substituents is not the right approach to improve the trypanocidal activity of the ionophore. Perhaps, other modifications of the carboxyl group like esterification or amidation could afford derivatives of lasalocid acid with enhanced antitrypanosomal activity as has recently been shown for salinomycin (Steverding et al. 2016).

This study confirms previous findings that polyether ionophore antibiotics are promising antitrypanosomal agents (Steverding and Sexton 2013, Steverding et al. 2016). Although lasalocid acid, studied here, was found to be less trypanocidal than salinomycin, it had a better selectivity and induced faster swelling than other ionophores. Lasalocid acid may be directly applicable for treatment of nagana disease particularly as the ionophore is used in cattle as medicated feed additive (Bovatec®). As no published data are available, it remains to be shown whether lasalocid acid supplementation can generate high enough plasma levels of the ionophore within its effective concentration range in cattle. However, when chickens were fed with 75 mg sodium lasalocid per kilogram of feed for 1 week, the mean concentration of the antibiotic in serum was 1.36 μg/ml (= 2.3 μM) (Stipkovits and Juhász 1987) which is above the GI_50_ value of 1.75 μM for the trypanocidal activity of the ionophore (see above). Even if lasalocid acid as feed additive does not provide high enough plasma levels in cattle to affect substantially trypanosomes, the use of the ionophore in food could have a positive impact on the efficacy of the drugs currently employed to treat nagana disease. On the other hand, higher blood levels of lasalocid acid can be achieved by intravenous administration. For instance, intravenous injection of 5 mg of sodium lasalocid per kilogram body weight in a dog resulted in blood levels of the ionophore of > 7.3 μg/ml (> 12 μM) for the following 30 min (Brooks et al. 1975), a concentration that killed trypanosomes in our in vitro assay (MIC = 10 μM, see above). In addition, at normal dosage, lasalocid acid is of low toxicity and usually causes no adverse side effects in cattle. Only in cases of overdosage, cattle show signs of acute intoxication which include anorexia, dyspnoea, tachycardia, ataxia and diarrhoea. Taken together, this information warrants investigations into the in vivo trypanocidal efficacy of lasalocid acid.

## Electronic supplementary material

Supplementary Table S1 (DOCX 39 kb).
