# Supplementary material for: Trypanosoma brucei: trypanocidal and cell swelling activities of lasalocid acid
Source: Parasitol Res. 2017 Sep 27;116(11):3229–33. doi: 10.1007/s00436-017-5624-6 (PMC5660140; doi:10.1007/s00436-017-5624-6)
Supplement: Supplementary file 1 — (DOCX 39 kb). [file 436_2017_5624_MOESM1_ESM.docx]

**Supplementary Table S1** GI_50_ and MIC values and ratios of lasalocid acid and seven Mannich base derivatives for *T. brucei* and HL-60 cells

|  | | | | | | | |
| --- | --- | --- | --- | --- | --- | --- | --- |
| Compound | R | *T. brucei* |  | HL-60 |  | Selectivity |  |
|  |  | MIC (μM) | GI_50_ (μM)^a^ | MIC (μM) | GI_50_ (μM)^a^ | MIC ratio | GI_50_ ratio |
| Lasalocid acid |  | 10 | 1.73±0.79 | 100 | 24.7±2.4 | 10 | 14.3 |
| **1** |  | 10 | 3.08±0.08 | 100 | 16.1±7.7 | 10 | 5.2 |
| **2** |  | 10 | 3.17±0.16 | 100 | 28.9±8.6 | 10 | 9.1 |
| **3** |  | 10 | 2.85±0.21 | 100 | 24.7±11.8 | 10 | 8.7 |
| **4** |  | >100 | >100  (27%)^b^ | >100 | >100  (0%)^b^ | 1 | 1 |
| **5** |  | >100 | >100  (17%)^b^ | >100 | >100  (0%)^b^ | 1 | 1 |
| **6** |  | 100 | 3.28±0.21 | 100 | 32.8±2.5 | 1 | 10.0 |
| **7** |  | 10 | 3.10±0.19 | 100 | 30.1±0.9 | 10 | 9.7 |

^a^ Mean values ± SD of three experiments

^b^ Values in brackets refer to growth inhibition at 100 µM (the highest concentration tested)
